# Unique Pathological Findings of Polarized Calcium Oxalate Crystals in Spondylodiscitis of an ESRD Hemodialysis Patient: A Case Report

**DOI:** 10.1002/ccr3.9709

**Published:** 2024-12-06

**Authors:** Parisa Mehrasa, Sepideh Hadimaleki, Nadia Shafiee, Amirreza Khalaji

**Affiliations:** ^1^ Department of Pathology Tabriz University of Medical Sciences Tabriz Iran; ^2^ Department of Pathology, Imam Reza Hospital Tabriz University of Medical Sciences Tabriz Iran; ^3^ Immunology Research Center Tabriz University of Medical Sciences Tabriz Iran; ^4^ Connective Tissue Diseases Research Center Tabriz University of Medical Sciences Tabriz Iran

**Keywords:** calcium oxalate crystals, end‐stage renal disease, hemodialysis, spondylodiscitis

## Abstract

Patients with end‐stage renal disease (ESRD) undergoing chronic hemodialysis are at an increased risk of developing spondylodiscitis, an infectious condition affecting the vertebral column. In this case report, we present a 22‐year‐old male with ESRD, a history of hyperoxaluria, nephrolithiasis, and anemia, who developed spondylodiscitis. Notably, pathological examination of tissue samples obtained during surgical intervention revealed the presence of polarized calcium oxalate crystals within the lumbar laminae and spinal discs, a rare finding in this clinical context. The deposition of these crystals may have contributed to the development and progression of spondylodiscitis by creating an environment conducive to bacterial growth and infection. This case highlights the importance of comprehensive pathological assessment in ESRD patients with spondylodiscitis, as it may uncover uncommon manifestations that could have implications for disease management. Further research is necessary to elucidate the underlying mechanisms of this rare presentation and its impact on the clinical course and treatment of spondylodiscitis in ESRD patients undergoing hemodialysis.

**Summary**

- Detailed pathological examination is of utmost importance in patients with end‐stage renal disease and concurrent spondylodiscitis.
- It can uncover rare findings, such as the deposition of polarized calcium oxalate crystals, which may significantly impact disease management.

## 1 Introduction

Patients with end‐stage renal disease (ESRD) undergoing chronic maintenance hemodialysis are at an increased risk of bloodstream infections. This elevated risk is attributed to the dialysis water purification system, repeated vascular punctures, or infected vascular access sites. Additionally, immune dysregulation in the uremic environment can impair the body's defenses against microbial invasion [1, 2, 3]. Spondylodiscitis refers to infectious conditions affecting the vertebral column. The incidence of spondylodiscitis has demonstrated a continuous increase in recent years [4]. Hemodialysis patients are at increased risk of bacteremia and its potential complication, spondylodiscitis. Previous studies have reported an annual incidence of spondylodiscitis ranging from 1 in 80 to 1 in 215 cases among the hemodialysis population [5].

Microbiology features, clinical manifestations, and results can vary in chronic dialysis cases compared to their non‐dialysis counterparts. Just several studies on dialysis patients showed spondylodiscitis [6, 7, 8]. Oxalosis also points to the deposition of calcium oxalate crystals in various tissues and organs, primarily resulting from Aspergillus infection [9]. Previous research has demonstrated the occurrence of these crystal deposits in the sinus, nasal cavity [9], and kidneys [10]. However, no studies have investigated their presence in intervertebral discs. In this case report, we present a patient with ESRD undergoing regular dialysis who developed spondylodiscitis. Notably, pathological examination revealed polarized calcium oxalate crystal deposition in the lumbar lamina and discs, an uncommon finding in such cases.

## 2 Case History/Examination

The patient is a 22‐year‐old male with a history of ESRD for the past 2 years due to hyperoxaluria, nephrolithiasis, and anemia. He has been receiving regular hemodialysis therapy three times per week. The patient presented to the clinic with complaints of general weakness, fatigue, and back pain. Physical examination revealed pale conjunctivae, lower extremity edema, and tachypnea. Therefore, the patient was admitted to the inpatient ward. The patient's medical management included folic acid, calcium, vitamin B6, and Renagel treatment.

## 3 Methods (Differential Diagnosis, Investigations, and Treatment)

The patient presented with suspected deep vein thrombosis (DVT) and underwent a Doppler ultrasound examination in the inpatient ward. The ultrasound findings did not indicate the presence of a blood clot. The patient then underwent a cardiac echocardiogram, which revealed the following findings: ejection fraction (EF) = 40%, left ventricular aneurysms (LVA), and mild left ventricular enlargement (LVE). Based on these findings, the patient was diagnosed with heart failure (HF) and subsequently received medical treatment.

Subsequently, the patient underwent an MRI evaluation, which revealed spondylodiscitis at the L5/S1 level and an epidural collection at the L5 level. Based on these findings, the patient was scheduled for surgical intervention and medical management (Table 1) with antibiotics was initiated for him. In addition, routine laboratory tests were performed, and the results are summarized in Table 2.

**TABLE 1:** Pharmacological management during hospitalization.

Pharmaceutical agent	Posology and administration route
Pantoprazole	40 mg per os (tablet)
Heparin	5000 IU intravenously for 10 days
Calcium	500 mg per os (capsule) for 10 days
Folic acid	5 mg per os (tablet)
Amoxicillin	500 mg per os (tablet)
Sevelamer (Renagel)	800 mg per os (tablet)
B‐Complex	per os (tablet)
Cyanocobalamin (vitamin B12)	per os (tablet)
Vancomycin	1 g intravenously, thrice every 5 days
Ceftazidime	1 g intravenously, single dose
Meropenem	1 g intravenously for 4 days
Rifampin	600 mg per os (capsule) for 3 days
Cefazolin	2 g intravenously for 4 days

**TABLE 2:** Laboratory parameters of the patient.

Laboratory parameters	Patient's values	Normal range
Leukocyte count, per μL	9.1 × 10^3^	4–10 × 10^3^
Hemoglobin, g/dL	5	12.3–15.3
Platelet count, per μL	17,700	150,000–450,000
INR, Index	1.05	0.9–1.0
PTT, s	37	25–45
CRP, mg/L	130	< 6
SGOT, g/dL	22	8–35
SGPT, g/dL	12	8–35
Alk. P, U/L	620	64–306
BUN, mg/dL	146	7–20
Creatinine, mg/dL	6.4	0.5–1.1
Sodium, mEq/L	128	136–145
Potassium, mEq/L	5.6	3.7–5.5
HBsAg	Negative	—
LDH, U/L	442	135–225
Ferritin, ng/mL	20	20–300
TIBC, μg/dL	195	250–450
Wright test	Negative	—
Coombs Wright test	Negative	—

Abbreviations: ALP, alkaline phosphatase; BUN, blood urea nitrogen; CRP, C‐reactive protein; HBsAg, hepatitis B surface antigen; INR, international normalized ratio; LDH, lactate dehydrogenase; PTT, partial thromboplastin time; SGOT, serum glutamic‐oxaloacetic transaminase; SGPT, serum glutamic‐pyruvic transaminase; TIBC, total iron‐binding capacity.

## 4 Conclusion and Results (Outcome and Follow‐Up)

**FIGURE 1:**
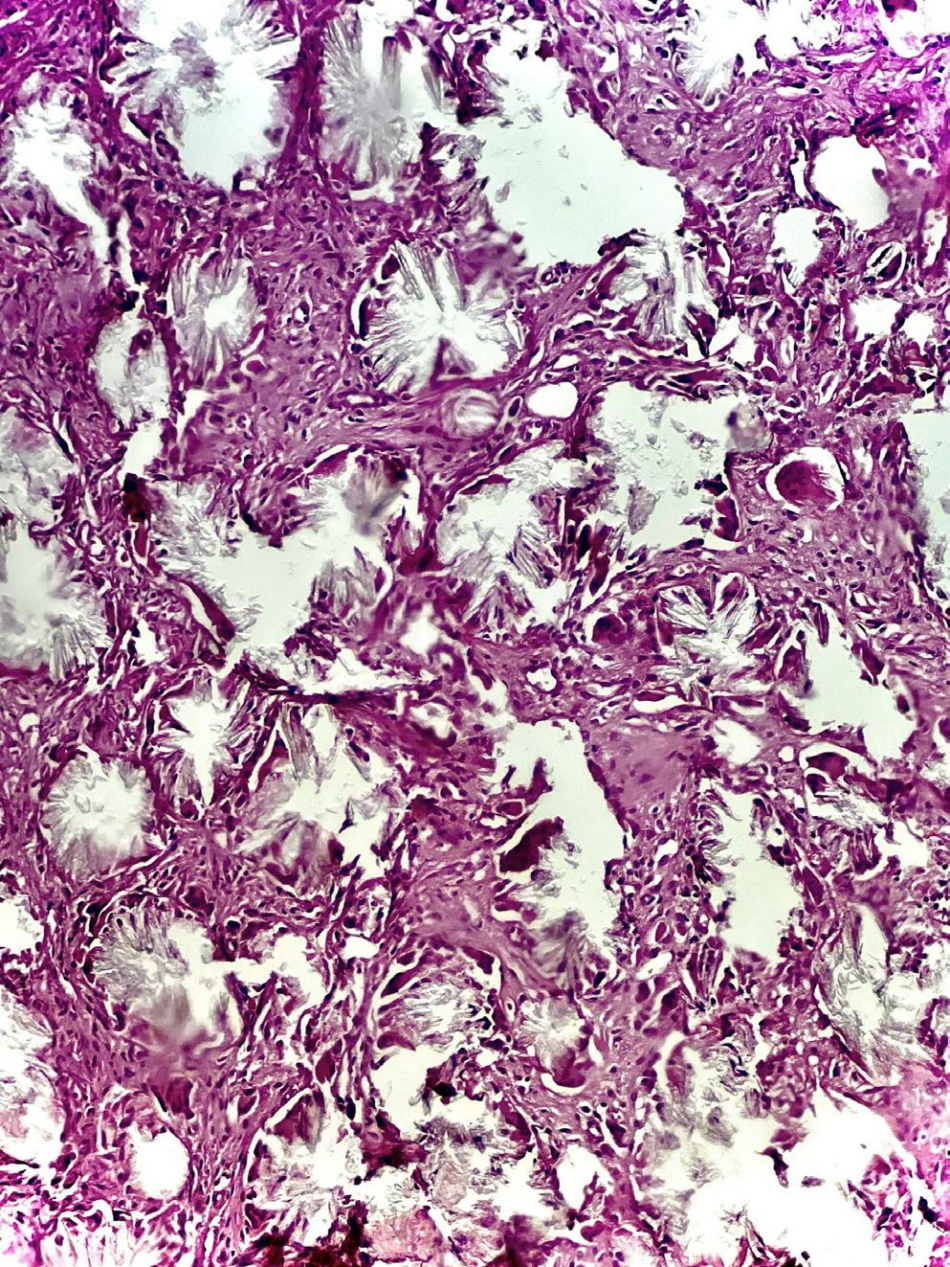
Hematoxylin and eosin (H&E)‐stained calcium oxalate crystals under light microscopy.

**FIGURE 2:**
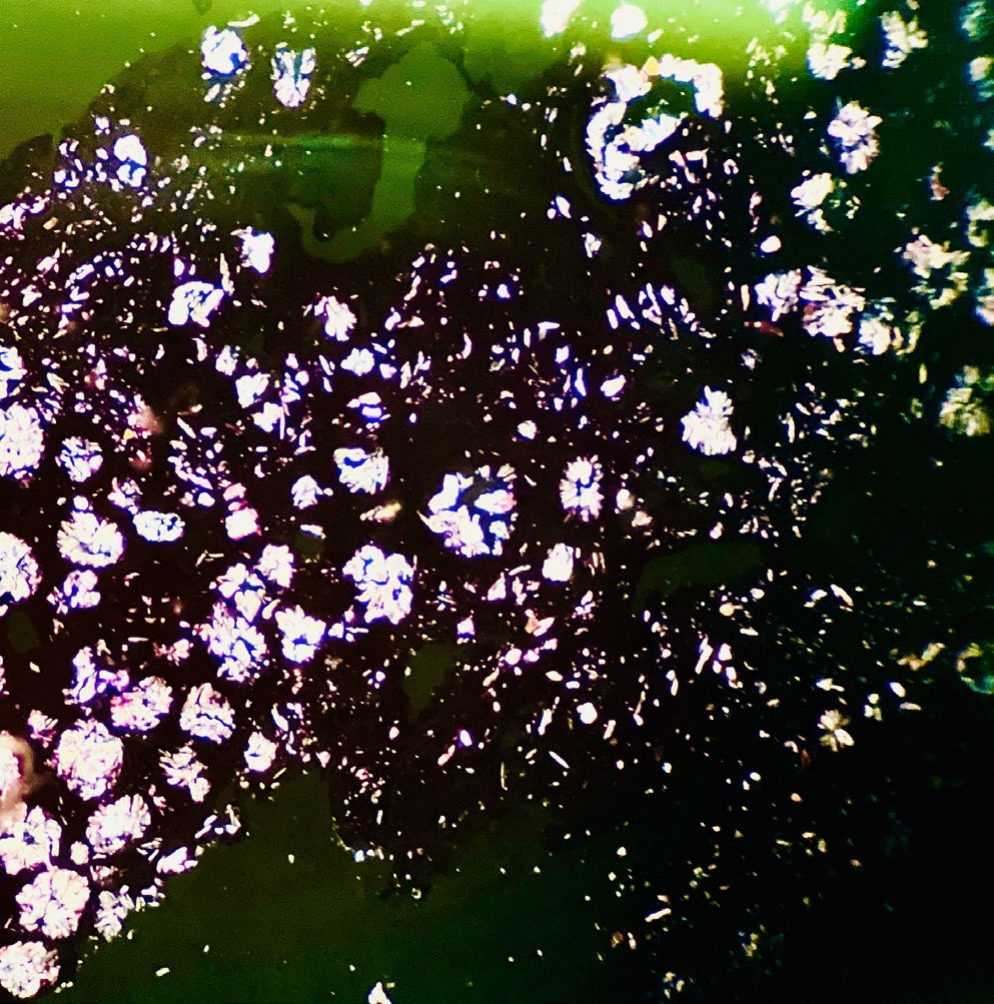
Hematoxylin and eosin (H&E)‐stained calcium oxalate crystals under polarizing microscopy.

The patient then received an L5/S1 laminectomy, an L5/S1 discectomy, and a decompression of the spinal canal surgery. Tissue samples acquired during the surgical intervention were sent for pathological analysis. The pathological evaluation (Figures 1 and 2) of the tissue samples obtained during the surgical procedure revealed the following:

1. Lumbar lamina; laminectomy: The tissue showed fibrous connective tissue with mild inflammation, abscess formation, and a foreign body type reaction associated with the deposition of polarized calcium oxalate crystals.
2. Disc L5‐S1; resection: The tissue exhibited fibrous connective tissue with mild inflammation and the deposition of polarized calcium oxalate crystals.

The occurrence of these crystals within the lumbar laminae and spinal discs is uncommon.

## 5 Discussion

This case report describes the rare occurrence of polarized calcium oxalate crystals found within the lumbar laminae and spinal discs of a hemodialysis patient diagnosed with spondylodiscitis. This represents an unusual and previously unreported pathological finding in this clinical setting. It underscores the significance of pathological examinations in ESRD cases, potentially impacting clinical management strategies. The case report's comprehensive structure, including detailed clinical history, methods, and discussion, provides valuable insights for nephrologists and infectious disease specialists, prompting further investigation into similar cases.

The detection of polarized calcium oxalate crystals within the lumbar laminae and spinal discs of ESRD cases with spondylodiscitis is a rare and previously unreported pathological finding in this clinical setting. These crystal deposits within the tissues are probably related to the patient's history of anemia, nephrolithiasis, and hyperoxaluria, all of which are associated with elevated oxalate levels [11, 12]. The formation of crystals may create an environment that is not only conducive to infection and bacterial proliferation but also potentially promoting spondylodiscitis development, underscoring the urgent need for further investigation.

Sharma et al. [13] reported two individuals with a rare presentation of bone marrow (BM) calcium oxalate crystal deposition. This condition resulted in recurrent nephrolithiasis and BM failure; both cases exhibited varying degrees of BM failure severity and recurrent renal stones. Furthermore, another case report demonstrated a destructive arthropathy affecting the shoulders and hips in a patient with tumoral calcinosis, which was associated with calcium oxalate deposits in a case of ESRD with primary oxalosis on hemodialysis [14].

Spondylodiscitis is a potentially severe complication associated with hemodialysis therapy. Its nonspecific clinical presentation, coupled with an insidious and gradual onset, often results in delayed diagnosis. In the context of hemodialysis patients, the recent onset of spinal pain may serve as a sentinel indicator of emerging spondylodiscitis [11]. Immunosuppression, prior spinal surgery, uncontrolled diabetes mellitus, infective endocarditis, and catheter‐associated infections represent well‐established risk factors for the development of spondylodiscitis among individuals undergoing hemodialysis. Existing studies have elucidated the clinical presentation of spondylodiscitis in this patient population, revealing that affected hemodialysis recipients, particularly those with comorbid diabetes or central venous catheters, may present with headaches, nausea, mild spinal stiffness, general malaise, or localized spinal pain. In contrast, non‐hemodialysis individuals with spondylodiscitis more commonly exhibit low back pain as the primary symptom [7, 15, 16].

The ESRD condition significantly impacts both adaptive and innate immunity. In ESRD, all variables of innate immunity are affected, ranging from the cellular receptors' response to various stimuli to dysregulated cytokine production and cellular dysfunction. Regarding adaptive immunity, ESRD is associated with dysfunction of antigen‐presenting cells, diminished B lymphocyte counts, and impaired activation of T lymphocytes [1, 2, 17]. The pro‐inflammatory states can be correlated with various pathological conditions, such as superimposed malnutrition, chronic infections, back filtration, endotoxin exposure, dialysate, bioincompatible dialysis membranes, and fistula or catheter‐associated infections [18]. Additionally, due to blood loss and erythropoietin deficiency, anemia is prevalent among this population. Consequently, common inflammatory biomarkers, such as C‐reactive protein and erythrocyte sedimentation rate, are routinely evaluated in hemodialysis patients even in the absence of any clear etiology [19], as they can provide prognostic information regarding potential underlying causes and cardiovascular mortality risk [20, 21].

Moreover, Ratiu et al. [22] showed that as infectious spondylodiscitis is associated with the presence of a central venous catheter, hemodialysis patients with arteriovenous fistulas, accounting for approximately 80% of this population, may exhibit a lower incidence of infectious spondylodiscitis. The accurate diagnosis is confirmed by correlating elevated inflammatory marker levels, clinical manifestations, and imaging‐based findings.

The limitations of this single case report include its limited generalizability to the broader ESRD population, necessitating more extensive cohort studies to determine the prevalence and clinical significance of calcium oxalate crystal deposition in spondylodiscitis among ESRD patients. Unidentified confounding factors, such as undiagnosed metabolic disorders or medication interactions, may have influenced crystal formation. Additionally, there needs to be long‐term follow‐up data, which restricts our understanding of disease progression and the implications of crystal deposition.

## 6 Conclusion

The rare deposition of polarized calcium oxalate crystals within the lumbar laminae and discs of this ESRD patient on hemodialysis who developed spondylodiscitis represents a unique pathological finding in this clinical context. These crystals may have contributed to the development and progression of spondylodiscitis by creating an environment conducive to bacterial growth and infection. This case highlights the importance of thorough pathological examination in ESRD patients with spondylodiscitis, as it can uncover rare manifestations that may have implications for management. Further research is needed to elucidate the mechanisms underlying this rare presentation and its impact on the clinical course and treatment of spondylodiscitis in ESRD patients on hemodialysis.

## Author Contributions

**Parisa Mehrasa:** investigation, writing – original draft, writing – review and editing. **Sepideh Hadimaleki:** data curation, resources, software, supervision. **Nadia Shafiee:** visualization, writing – original draft. **Amirreza Khalaji:** conceptualization, resources, validation, visualization.

## Ethics Statement

This study was performed according to the principles outlined by the World Medical Association's Declaration of Helsinki on experimentation involving human subjects, as revised in 2000, and has been approved by the ethics committee of the Tabriz University of Medical Sciences.

## Consent

The patient gave written informed consent to publish this report and clinical images. The consent has been signed and collected in accordance with the journal's patient consent policy.

## Conflicts of Interest

The authors declare no conflicts of interest.

## Data Availability

The research data used in this study are available from the corresponding author upon reasonable request.
